# A working hypothesis visualization method for fNIRS measurements using Monte Carlo simulation

**DOI:** 10.1016/j.mex.2023.102357

**Published:** 2023-09-09

**Authors:** Yota Kikuchi, Yasutomo Nomura

**Affiliations:** Department of Systems Life Engineering, Maebashi Institute of Technology, Maebashi 371-0816, Japan

**Keywords:** A working hypothesis visualization method for fNIRS measurements using Monte Carlo method, Optical properties, Standard brain, Colin27, MNI152, MNI space, Finger tapping, Automated anatomical labeling, Brodmann area

## Abstract

In neuroscience, clarifying the functional localization of the cerebrum using functional near-infrared spectroscopy (fNIRS) is one of the important works. To better understand and trust fNIRS data, neuroscientists formulate hypothesis about the underlying neural processes. However, visualizing and validating these hypotheses is not easy due to the complex nature of brain activity and the limitations of fNIRS measurements. In this paper, we suggest the novel Monte Carlo tool designed to assist fNIRS study for neuroscientists and to deal with these problems. The tool provides a user-friendly interface for generating realistic virtual brain activity patterns based on a specified hypothesis. By setting up a region of interest in the standard brain based on the hypothesis, the simulation models the propagation of light through the brain accurately and mimics the hemodynamic response observed in fNIRS measurements. By visually displaying simulation data and identifying the major activation point, neuroscientists can validate and refine hypothesis and obtain a better understanding of the neural mechanisms underlying the fNIRS signals.

• A Monte Carlo simulation method reflecting the functional localization of the cerebrum for fNIRS measurements.
• Method for setting ROI corresponding to the functional localization of the cerebrum in the standard brain.
• Visualization of Monte Carlo simulation results and anatomical evaluation method of activation points.

## Graphical abstract

**Figure:**
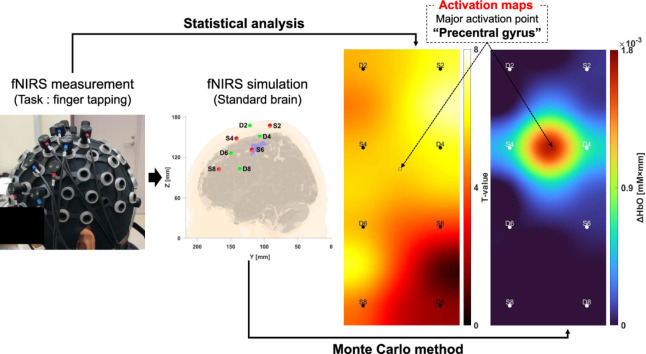

Specifications Table

**Table:** 

Subject area:	Neuroscience
More specific subject area:	Neuroscience methodology
Name of your method:	A working hypothesis visualization method for fNIRS measurements using Monte Carlo method
Name and reference of original method:	Not applicable
Resource availability:	LIGHTNIRS, Monte Carlo eXtreme, NIRS-SPM, 3D digitizer

## Method details

### Background

The functional near-infrared spectroscopy (fNIRS) is a method for non-invasive measurement of brain activity [1]. The fNIRS has been used to measure the brain activity for a variety of tasks and is becoming established as a method for mapping brain function. In the past, when neuroscientists reported on unknown neural networks, they have no choice but to use analog images and conceptual diagrams to explain. These lead to ambiguity in explanations for neural networks. In addition, when using fNIRS for clarifying the neural networks, it is necessary to predict active regions from previous reports and original hypothesis before the fNIRS measurements. A more reliable result requires an approach based on solid evidence and experimental data. Therefore, we think that it is meaningful in the neuroscience field to approach fNIRS measurements with a working hypothesis in advance and to have a solid comparison criterion for validating the measured fNIRS data.

In this study, we developed a method for visualization of the working hypothesis using Monte Carlo simulation in fNIRS measurements and a method for anatomical evaluation of activation points. This method allows neuroscientists to visualize the virtual brain activity before performing fNIRS measurements. Therefore, a smoother approach to actual measurement can be achieved. In addition, by performing Monte Carlo simulation that mimic actual fNIRS measurements, we can generate theoretical evaluation criteria and confirm the validity of measured data. This makes it possible to objectively evaluate the validity of the measurement results. These will increase the reliability of the data and lead to the reporting the high-quality results in academic papers. The purpose of this paper was to demonstrate that the visualization method of working hypotheses using Monte Carlo simulation for fNIRS measurements is a useful and robust tool. Therefore, in this paper, our work was to compare the fNIRS data that was actually measured and the simulation results that mimicked the fNIRS measurement.

## fNIRS measurement

### Experimental protocol

**Fig. 1:**
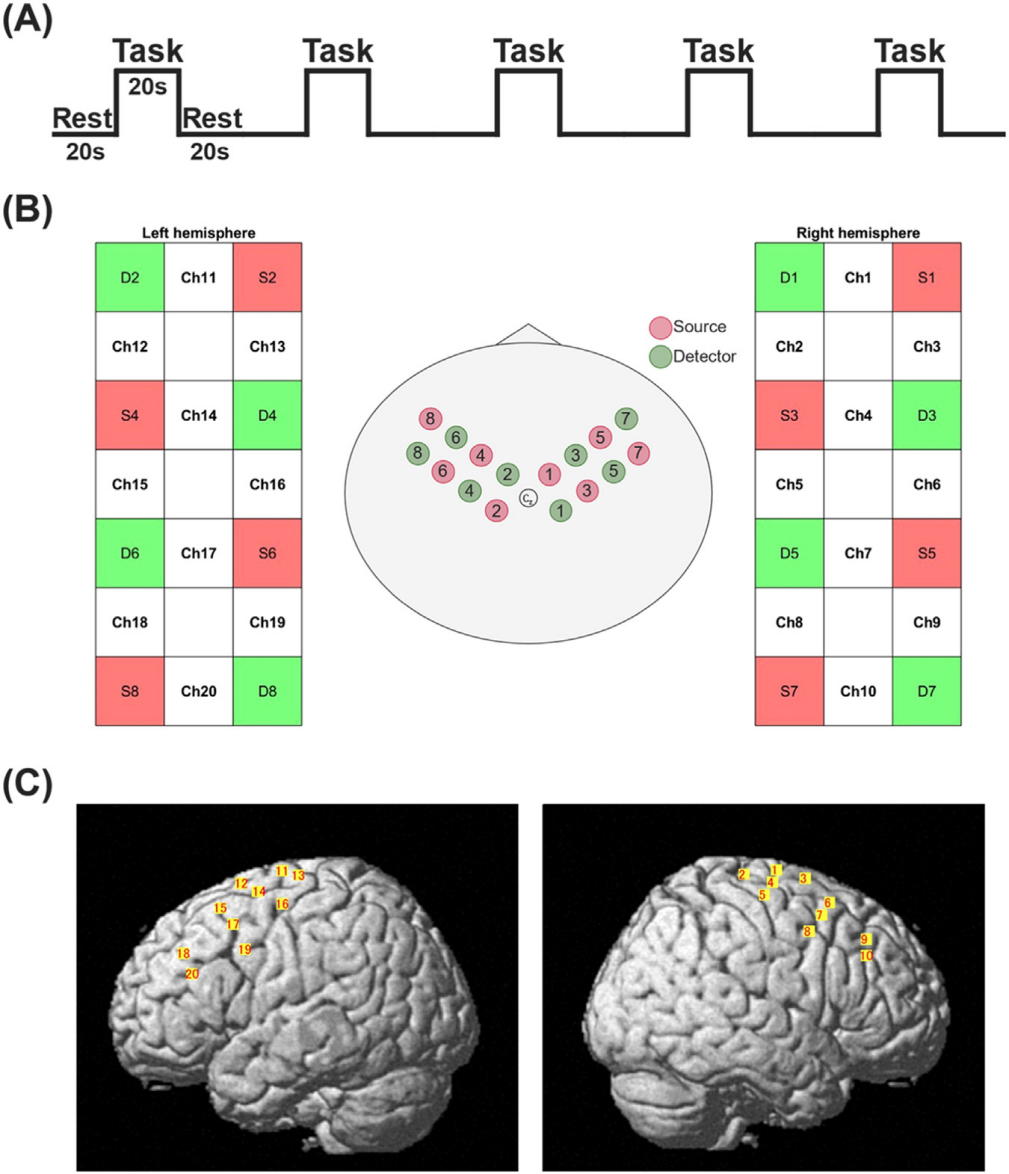
Study design. (A) Experimental paradigm. (B) fNIRS optode layout design. (C) Lateral view of the corresponding anatomical regions of channel configuration.

Finger tapping, which is a basic task in many papers, was adopted as the measurement task in this study [2], [3], [4]. Finger tapping has the advantage of eliminating movements other than fingertips. Furthermore, it is thought that the activation area tends to appear remarkably and stably because the measurement can be performed in a relaxed state with little emotional fluctuation. In this study, finger tapping was performed by tapping the thumb and index finger. Tapping task was performed with the left and right hands in sequence. Fig. 1(A) shows the paradigm design. Protocol began with 20 s rest period as a baseline. Afterwards, finger tapping was performed for 20 s. After each tapping segment, rest period was provided for 20 s, and this cycle was repeated 5 times. Two sets of experiments were performed with the right and left hands, respectively. Also, tapping movement was performed at a pace of approximately 1 Hz during the task period. At this time, the computer monitor displayed a signal to start and finish the task, and the subject changed their behavior according to the signal.

### Instrumentation

Portable optical brain imaging device for research (LIGHTNIRS, SHIMADZU Corporation, Japan) was used to measure the cortical activity. The device had eight near-infrared semiconductor lasers and eight avalanche photodiode detectors. Each source emitted at three wavelengths (780, 805, 830 nm). The relative concentration changes of oxy-hemoglobin (HbO), deoxy-hemoglobin (HbR), and total-hemoglobin (HbT) were measured as time series signals at a sampling rate of 13.33 Hz based on the modified Beer–Lambert law. The change in the concentration of HbO can be represented as in Eq. (1) from the extinction coefficients for each wavelength [5,6].

(1) $$\Delta \text{HbO}\;=\;-1.4887\;\times \;\Delta A_{\left(780\right)}+\;0.5970\;\times \;\Delta A_{\left(805\right)}\;+\;1.4847\;\times \;\Delta A_{\left(830\right)}.$$

Δ$A_{\left(\lambda \right)}$ indicates changes in light absorption at the corresponding wavelength, i.e., the measured value for each wavelength, can be represented as in Eq. (2).

(2) $$\Delta A_{\lambda }=-\log_{10}\frac{I_{out}+\Delta I_{out}}{I_{out}},$$

where, $I_{out}$ is a detected light intensity and Δ$I_{out}$ is an amount of changes in detected light intensity.

In this study, we used a cap-type holder in which probe mounting positions are arranged at intervals of 3 cm. As shown in Fig. 1(B), assuming activation of the precentral gyrus (primary motor cortex) by finger tapping, probes were placed in each hemisphere centered on Cz based on the international 10–20 system. Four sources and detectors were placed alternately. The middle points between the source and the detector were called “NIRS channel”, and signals from a total 20 channels were detected in this study. The NIRS signals from the detector are thought to reflect hemodynamics under these channels. When attaching the holder, the hair was moved out of the way at the probe position to reduce the noise caused by hair. In addition, the subject's head was covered with a special light-shielding cover to reduce the ambient light that might be detected.

The spatial coordinates of the four reference points (Nz, Cz, AL, AR) based on the international 10–20 system and 16 optical probes were recorded precisely using the 3D digitizer (FASTRAK, Polhemus, USA). These coordinates were transformed to position of 20 channels and 16 probes in the Montreal Neurological Institute (MNI) space using NIRS-Statistical Parametric Mapping software (NIRS-SPM) [7,8]. This is called “probabilistic registration”. Each NIRS channel and probe were located on the surface of the standard brain (MNI152). Through this transformation, the maximum likelihood estimates of the position of NIRS channels and probes in Automated Anatomical Labelling (AAL) or Brodmann area were computed. Fig. 1(C) shows the result of probabilistic registration in each cerebral hemisphere. Based on the AAL, the ch2, ch4, ch8, ch11, ch13, ch14 and ch19 monitored for precentral gyrus activity. Middle frontal gyrus activity was monitored in ch6, ch7, ch9, ch10, ch15, ch17 and ch18. Furthermore, superior frontal gyrus activity was monitored in ch1, ch3, and ch12. The ch5 and ch16 were located on the postcentral gyrus and ch20 was located on the interior frontal gyrus. Not all channels were placed on the precentral gyrus due to the nature of the holder used.

### Participant and preparation

In this study, fNIRS data were collected from one healthy adult (male; age = 60 years). The subject is right-handed and had no history of mental or neurological disorders that might affect measurement. The study was approved by the local ethics committee of the Maebashi Institute of Technology (23-006) and the subject gave informed consent before the experimental sessions. The subject sat on a chair during data collection and was asked to remain as still as possible and focus on the computer monitor.

### fNIRS data analysis

NIRS-SPM was used for analysis of the fNIRS data [7]. In most recent fNIRS study, HbO has been used as the primary index of cortical activation. This is because HbO is the most sensitive index of regional cerebral blood flow changes [9,10]. Therefore, the HbO level was used as a marker of cortical activity in this study as well. Although optical probes were placed on a right and left head surface during the fNIRS measurement, only fNIRS data obtained from the cerebral cortex contralateral to the tapping hand were used in the analysis. This was because cortical activity in motor tasks could be expected to occur in the hemisphere contralateral to the input.

The internal routine of NIRS-SPM was adopted for pre-processing algorisms. Wavelet-MDL (minimum description length) detrending algorism was used as a high-pass filter to remove global trends due to respiration, heart rate, vasomotion, and other experimental errors [11]. In addition, HbO signals of each channel were processed by hemodynamic response function (HRF) precoloring method and high-frequency noises were removed [12]. Next, the signals were analyzed based on general linear model (GLM) using a canonical hemodynamic response curve. GLM was calculated by modeling the HbO responses of hypothesis for the task condition using the default settings of NIRS-SPM. GLM analysis was performed to test for significant cortical activation under the experimental conditions. Statistical contrasts with reference to the baseline signal were tested, and cortical activity during the task was represented as *t*-values. The *p*-value was set to within 0.05 to obtain a clear result. Finally, *t*-statistic map was plotted on the standard brain (MNI152) to visualize areas of significant contrast in HbO concentrations.

## Monte Carlo simulation

Monte Carlo method has been widely used for more than 20 years to simulate light propagation in biological tissues because of their ability to strictly solve photon transport problems in heterogeneous media with complex structures [13]. Monte Carlo eXtreme (MCX) is the faster photon transport simulator using a huge number of parallel threads in the graphics processing unit (GPU) [14]. MCXLAB, a MATLAB version of MCX, was used in this study. It is offered as an open software package. All simulations were run in the MATLAB R2021a environment on a computer with 32 GB memory and an NVIDIA Quadro T2000 GPU.

### Setting ROI based on the functional localization of the cerebrum to 3D head model

In this study, Colin27 was used as the 3D head model for simulation [15]. Colin27 is a standard brain published from MNI. Colin27 has clearer brain structures than MNI152, which was used for fNIRS data analysis, and has often been used in Monte Carlo simulations for the head. In addition, it is registered in the MNI space and can identify the anatomical region by specifying (x, y, z) coordinates similar to MNI152. The simulations used a labeled Colin27 head voxel model available from MCXLAB. The size of the head voxel model was 181 × 217 × 181 mm (X × Y × *Z*), and the size of each voxel was 1 × 1 × 1 mm. In addition, the model has been labeled as air, scalp, skull, CSF, gray matter, white matter.

The users who will use the tool presented in this paper can accurately simulate virtual brain activity by setting the proper region of interest (ROI) in the head voxel model. For example, in the case of finger tapping like this study, it can be predicted that activity will appear significantly in some area of the precentral gyrus (the primary motor cortex) [2], [3], [4]. Since the precentral gyrus of the Colin27 was defined by AAL or Brodmann area, precentral gyrus of the head voxel model used in the simulation was identified from the previous reports [16,17]. In addition, we identified the regions that are particularly involved in finger movement within the precentral gyrus based on the Homunculus (Thumb, Index, Middle, Ring, and Little) [18]. This process was done by overlaying the functional localization image of Homunculus and the coronal slice image within the precentral gyrus of the head voxel model at the same scale. Finally, the identified regions were labeled as the seventh region using ImageJ, an image analysis software. This process was done by following steps. First, the head voxel model file was loaded into ImageJ. Next, the “Freehand selections” that exists on the ImageJ UI is used to enclose an arbitrary region. Finally, the program created by the ImageJ macro, which was provided in supplementary material (labeling.ijm) was run, so that identified region was labeled exactly. Note that fNIRS device measures the blood flow changes on the surface of the cerebral cortex, mainly changes in hemoglobin concentration in capillaries. It is reported that the capillaries are dense in the region up to a depth of 2 mm from gray matter [19]. Therefore, the ROI was set to a depth of 2 mm (two voxels) from the surface of the gray matter. As a result, the head voxel model was labeled as in Fig. S1.

### Probe conversion from the cerebral cortex surface to the scalp surface

The tool presented in this study employs two approaches for setting up the optical probe. One approach is manually placing the probes at positions determined by the user. This approach is useful for the visualizing brain activity based on the working hypothesis before the actual measurements. In this case, the process is simple: the coordinates of the scalp surface of the head voxel model selected by the user are set as the probe position. Another approach is reflecting the probe positions placed on a subject's head during actual measurement in the head voxel model. This approach is useful for the confirming the validity of fNIRS measurements against simulation results. Since this study aims clarifying the robustness of this tool by comparing results of actual measurement and simulation, the location of probes during fNIRS measurement was reflected to the head voxel model. In this paper, we described the method for probe placement on the scalp surface of the head voxel model (Colin27) based on the MNI coordinates obtained from the probabilistic registration of NIRS-SPM. First, the head voxel model used in this simulation was converted to the MNI coordinate space. The anterior commissure of the head voxel model was defined as the origin. The head voxel model was aligned to the MNI coordinate system by affine transformation. The line from the anterior commissure to the posterior commissure is the y-axis, and the line extending from the anterior commissure to the midline perpendicular to the y-axis is the z-axis. The x-axis is the normal to the yz-plane passing through the origin. This is the same as the general registration method to the MNI space. Next, a program was created in MATLAB to find the closest scalp surface coordinates to the MNI coordinates of each probe position obtained from the probabilistic registration by NIRS-SPM. The program was provided in supplementary material (probeconversion.m). This process is based on the assumption that the probe location of the cerebral cortex surface should be placed on the scalp closest to it. The voxel coordinates of the scalp surface of the head model were acquired and saved as text file (scalp_surface.txt) in advance. The distance between the two points of one probe coordinate and all scalp surface coordinates was calculated, and the scalp surface coordinate with the shortest distance between the two points were set as the probe position on the head voxel model. This process was done for all probes, so that probes located on the cerebral cortex surface were placed on the most proximal scalp surface (shown in Fig. S2).

### Simulation conditions

Table 1 shows the optical properties used in this simulation. Absorption coefficient $\mu_{a}$, scattering coefficient $\mu_{s}$, refractive index *n*, and anisotropy *g* was considered. It has been reported that the cerebral cortex activation increased HbO by 9 µM and decreased HbR by 3 µM [20]. Therefore, the absorption coefficient $\mu_{a}$ of the ROI was calculated according to the previous report [21]. The simulation was conducted for each source and detector pair, so that the fNIRS data of all channels were computed. Each source launched 100 million photons and the detector corresponding to the channels captured photons. At this time, the photons were launched toward the anterior commissure (MNI origin) in the head voxel model. This is based on the assumption that the MNI origin is approximately the center of a head. Since the photons once launched into the head voxel model occurred isotropic scattering event, we think that setting the incident vector does not lead to significant errors. Optical properties in each tissue at 780, 805, 830 nm based on Table 1 was sequentially assigned. Also, diameter of the detector is defined as 3 mm, which is the same as the fNIRS device (LIGHTNIRS). Each photon loses a part of weight in every photon-tissue interaction site, namely voxels. The absorption fluence is calculated by scoring weights in voxels. ΔHbO value of each channel in the simulation was determined by substituting the average value of absorption fluence value of the area set as the detector into Eqs. (1) and (2) as the detected light intensity. Note that the baseline simulation was conducted by setting the optical properties of the gray matter shown in Table 1 to the ROI. For example, considering the ch1, source number 1 and detector number 1 are placed on the head voxel model. First, baseline optical properties at 780 nm are set in the head voxel model to capture photons. The fluence value of the detector area is set as $I_{out}$. Next, activation optical properties at 780 nm are set in the head voxel model. The fluence value is obtained and is set as $I_{out}$ + Δ$I_{out}$. The $I_{out}$ and $I_{out}$ + Δ$I_{out}$ are substituted into Eq. (2). By conducting the same procedure at 805, 830 nm, ΔHbO of the ch1 computed by the simulation is obtained. A total of 120 simulations were performed to obtained ΔHbO for 20 channels in this study. The time required for one simulation was about 1 min and 30 s.

**Table 1:** Optical properties of each tissue and ROI.

Wavelength (nm)	Tissue	µ_a_ (mm^-1^)	µ_s_ (mm^-1^)	*n*	*g*
780	Air	0	0	1	1
	Skin	0.0208	17.2	1.45	0.89
	Skull	0.0158	14.5	1.45	0.89
	CSF	0.0042	2.53	1.33	0.89
	Gray matter	0.0367	21.2	1.45	0.89
	White matter	0.0163	84.3	1.45	0.89
	ROI	0.0374	21.2	1.45	0.89
805	Air	0	0	1	1
	Skin	0.0195	16.9	1.45	0.89
	Skull	0.0163	13.8	1.45	0.89
	CSF	0.0048	2.37	1.33	0.89
	Gray matter	0.0382	20.2	1.45	0.89
	White matter	0.0172	82.2	1.45	0.89
	ROI	0.0394	20.2	1.45	0.89
830	Air	0	0	1	1
	Skin	0.0191	16.3	1.45	0.89
	Skull	0.0168	13.5	1.45	0.89
	CSF	0.0055	2.14	1.33	0.89
	Gray matter	0.0408	19.2	1.45	0.89
	White matter	0.0181	80.6	1.45	0.89
	ROI	0.0423	19.2	1.45	0.89

In the Monte Carlo simulations, a seed value is required to generate random numbers. Changing the seed value also occurs the changing the fluence value. This effect may make inaccurate the results in this simulation, which must reflect the slightly absorption changes of cerebral cortex surface. Therefore, the same seed value was used for all simulations in this study. Since the random numbers generated are reproducible, photons that do not reach the ROI are not affected by changes in optical properties and travel in the same trajectory before and after activation. Only if the photon reaches the ROI, it will travel a different trajectory before and after activation. So, the fluence value can reflect slightly changes in absorption coefficient during activation.

## Method validation

Fig. S3 shows the *t*-statistic maps of fNIRS measurement calculated by NIRS-SPM. Fig. S3(A) shows the activation area of left cerebral hemisphere in right finger tapping. The *t*-values indicated that the most significant activation area was ch13 (*refer* Fig. 1(C)). In addition, Fig. S3(B) shows the activation area of right cerebral hemisphere in left finger tapping. The *t*-values indicated that the most significant activation area was ch3. The *t*-values of each channel are an important evaluation index for fNIRS data [7]. In the fNIRS study, a channel with the highest *t*-values is concluded to be the most active regions. However, we think that it is also important to evaluate the centroid of brain activity levels in the entire measurement area. Evaluating the centroid leads to viewing the tendency of activation in the area. It would be an important indicator for fNIRS with low spatial resolution. Therefore, the brain activity of the simulation and fNIRS measurement were evaluated by the centroid as evaluation index in this paper.

**Fig. 2:**
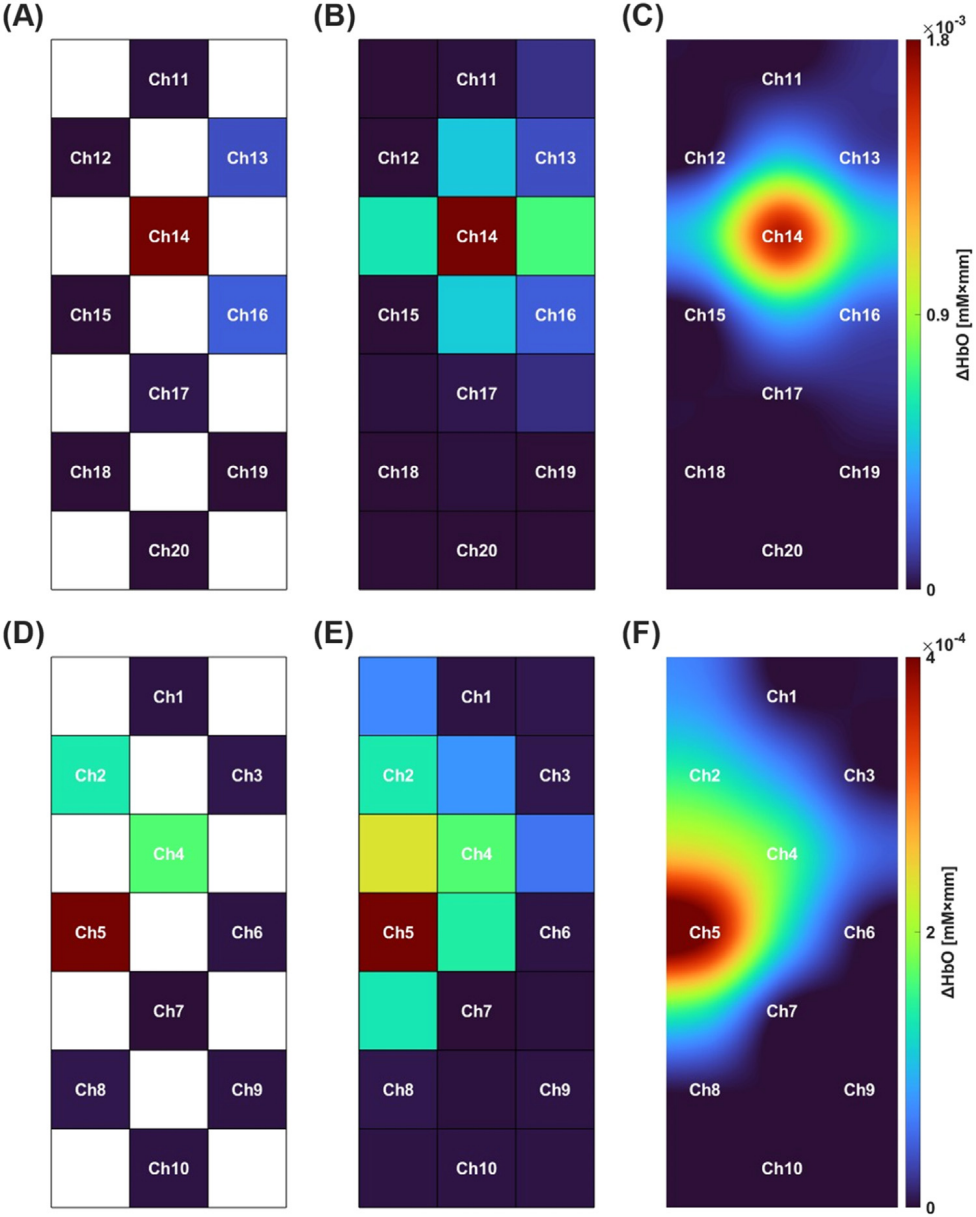
The activation map calculated by the simulation. (A) Mapping of calculated ΔHbO values for each channel in right finger tapping. (B) Linear interpolation result for blank areas of (A). (C) High-resolution 2D activation map with linear interpolation of all areas in (B). (D) Mapping of calculated ΔHbO values for each channel in left finger tapping. (E) Linear interpolation result for blank areas of (D). (F) High-resolution 2D activation map with linear interpolation of all areas in (E).

Fig. 2 shows the results of two-dimensional visualization of the intensity map of ΔHbO calculated by the simulation. The ΔHbO values calculated for each channel were applied to the corresponding positions in the 7 × 3 grid (shown in Fig. 2(A) and (D)). Next, ΔHbO values for the area where no data were applied were obtained by linear interpolation (shown in Fig. 2(B) and (E)). Finally, high-resolution two-dimensional activation colormaps were created by linearly interpolating the entire grid (shown in Fig. 2(C) and (F)). These results are useful for the users to visually identify the activation area of brain. The program for a series of processes is available in the supplementary material (activationmap.m).

Next, results of the fNIRS measurement and the simulation were compared. The ΔHbO values measured by fNIRS device provide the data with a noise component. This is due to the complex nature of brain activity, the differences in brain and head structure between individuals, and more. Therefore, noise is removed from the measured data by pre-processing, and uncertainty is reduced by performing statistical analysis. Furthermore, the activation maps created based on the analysis results are displayed on the standard brain, allowing for consistent discussion of anatomical regions. On the other hand, ΔHbO values calculated by the simulation do not contain noise components. In addition, we proposed the simulation method with a single standard brain in this study. Therefore, it may be inappropriate to simply compare ΔHbO in fNIRS measurements with ΔHbO calculated from the simulation. So, we compared the *t*-values calculated by the statistical analysis of the measured data and the ΔHbO values calculated by the simulation, and the major activation points were evaluated anatomically. In general, it may not be appropriate to compare values in different units. However, in the case of the simulation that solves the photon transport problems strictly and provides the results with extremely little fluctuate, such as in this study, we think that it is not a problem. The statistical analysis is unnecessary step for the simulation data. This would make the process smoother and less stressful for users.

**Fig. 3:**
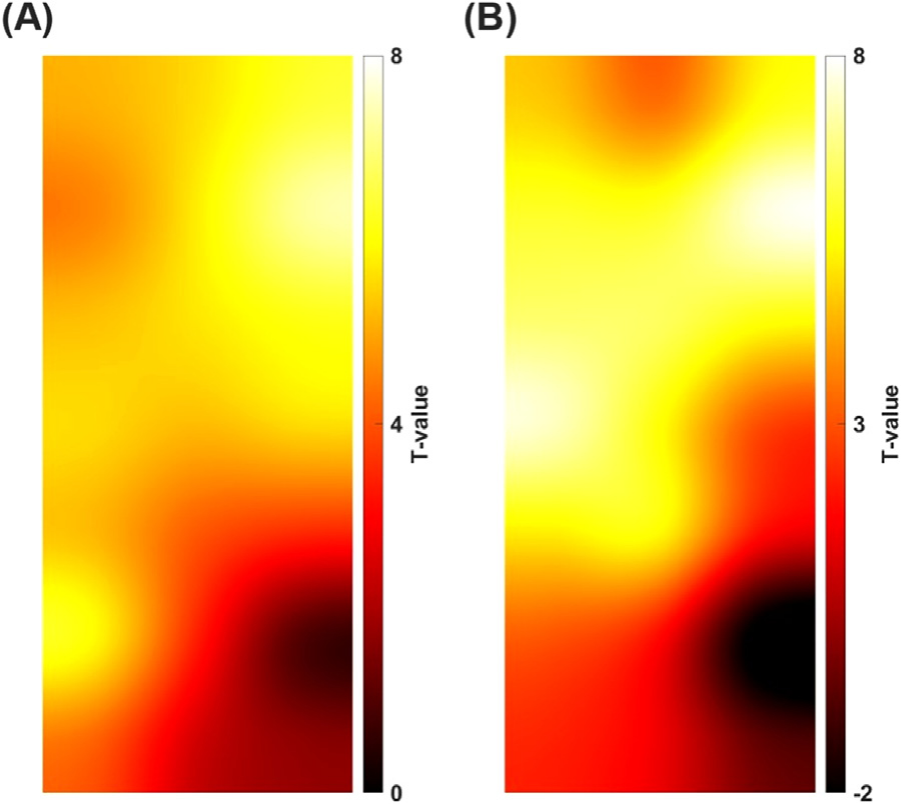
High-resolution 2D activation map estimated by *t*-statistical analysis (NIRS-SPM). (A) Activation map of the left cerebral hemisphere in right finger tapping. (B) Activation map of the right cerebral hemisphere in left finger tapping.

**Fig. 4:**
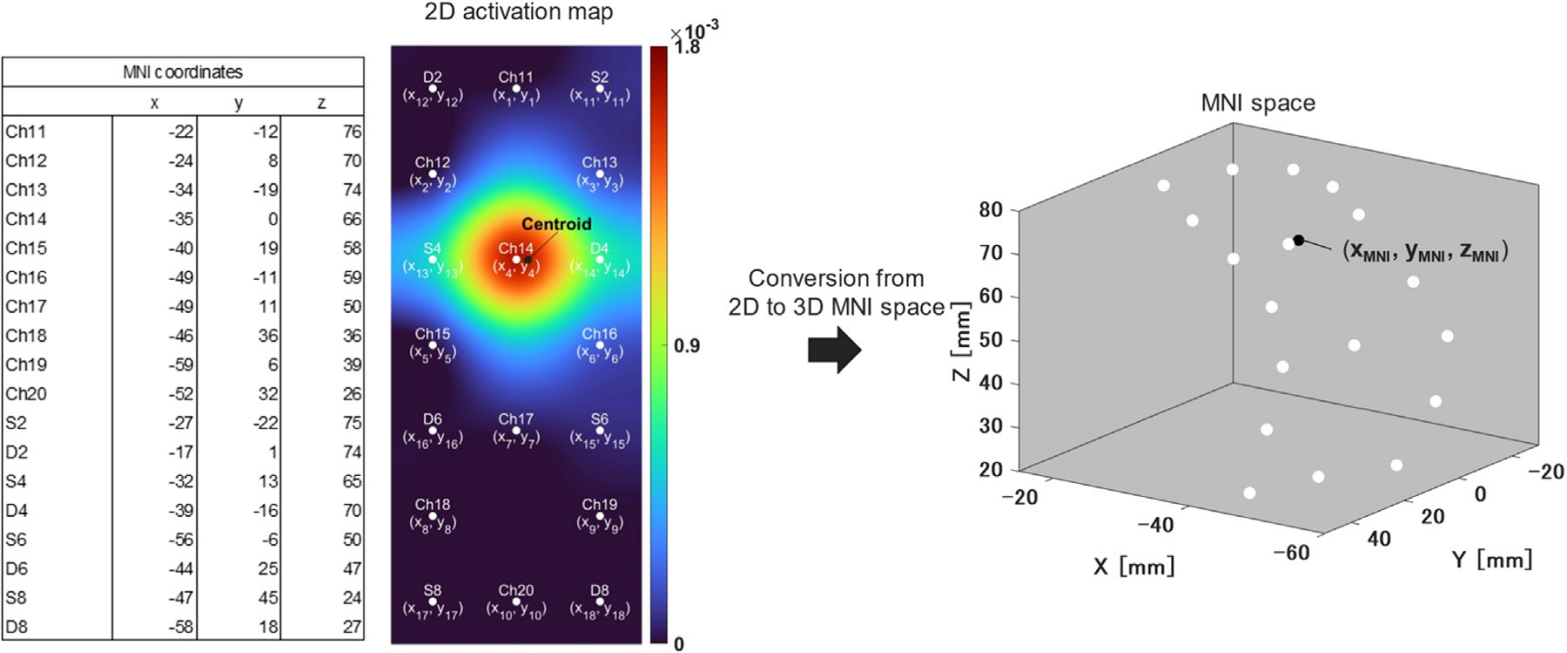
How to convert coordinates from 2D activation map to 3D MNI space (for right finger tapping simulation).

First, the *t*-values of each channel calculated by NIRS-SPM were reconstructed in two dimensions using the same method as in Fig. 2. In other words, this means that the activation map shown in Fig. S3 was reconstructed in two dimensions. Fig. 3A and B shows the activation map of the left cerebral hemisphere for right finger tapping and the right cerebral hemisphere for left finger tapping, respectively. Next, the program was created in MATLAB, which calculate the centroid of the 2D activation map (provided as activationmap.m). The calculated centroid was defined the major activation point of each activation map. Fig. S4 shows the centroid in each 2D activation map. We think that it is important for the neuroscience field to clearly indicate where the centroid is located in the anatomical region. Specifying the anatomical region of the centroid would facilitate comparisons between different studies and help generalize the results. Although the anatomical region is determined by MNI coordinates, note that the 2D activation maps as shown in Figs. 2 and 3 did not correspond to the MNI coordinates. As an example, Fig. 4 shows how to correspond the 2D activation map of a simulation in right finger tapping to the MNI space. The program was described in matlab code (activationmap.m). The coordinates (x, y) where each channel and probe located in the 2D activation map were overlayed with the MNI coordinates (x, y, z) of each channel and probe obtained from the probabilistic registration by NIRS-SPM. Therefore, the 2D activation map could be discussed in MNI space. The MNI coordinate of centroid was determined by three-dimensional interpolation of the scatter data using scatteredInterpolant function of MATLAB from the MNI coordinates of each point. The MNI coordinate of centroid was identified as the anatomical region based on the AAL by probabilistic registration function of NIRS-SPM. All 2D activation maps were transformed to the MNI space and the MNI coordinates of the centroid were calculated. As a result, both the centroids of the simulation and fNIRS measurement in right finger tapping were located in precentral gyrus, with a difference in centroid of 8.7 mm. Similarly for the left finger tapping, the centroids of the simulation and fNIRS measurement showed a misalignment of 7.7 mm, and both anatomical regions were determined to be precentral gyrus. We think that it is inevitable that the centroid position of the simulation results will differ from the actual measurement results, which measuring the unstable brain signals and estimating brain activity by statistical analysis. However, the difference between the centroid position of the simulation and the fNIRS measurement in this study was less than 10 mm, which we think is not a significant difference. First, the estimated accuracy of the probabilistic registration is reported to be 8 mm average standard deviation [8]. In other words, an error of about 8 mm occurs at the stage of reflecting the probe placement during fNIRS measurement to the standard brain. Also, since the width of the gyrus, an important functional unit of the brain, is about 10 mm, analysis at the level of the gyrus is possible. Furthermore, the calculated centroid, i.e., the major activation point, was located in precentral gyrus for both simulations and measurements. Although a misalignment of the centroid between simulation and actual measurements is inevitable, we think that it is feasible to visualize the virtual brain activity based on the working hypothesis for fNIRS measurements.

Since this tool uses the open software package only, there are no major restrictions on usage. Users will be able to customize the tool to suit their own research purposes (probe placement, ROI setting position, and more). By visualizing the working hypothesis before fNIRS measurement, it is possible to confirm the validity of the hypothesis and to refine the hypothesis. Since the optimal probe placement and measurement area can be determined in advance, fNIRS measurements can be performed efficiently. Also, by performing the simulation that mimics the actual fNIRS measurement as in this study, comparison with the actual measurement data is possible. In other words, simulations will provide data that support the experimental results. The brain activity in the actual measurement differs between subjects and trials. If the misalignment of the centroid between each measured data and a single simulation result is 10 mm or less, we think that the data to be more reliable.

## Conclusions

In this paper, we suggested the working hypothesis visualization method for fNIRS measurements using Monte Carlo simulation. To evaluate the robustness of the tool, fNIRS data for finger tapping task and the simulation data which mimicked the fNIRS measurement were compared. By setting the ROI at the position based on the hypothesis and reflecting the appropriate probe placement and hemodynamics in the simulation, it was confirmed that the results were in good agreement with the fNIRS measurement results. This tool will contribute to the clarification of the new neural network mechanisms.

## Ethics statements

This study was approved by the local ethics committee of the Maebashi Institute of Technology (23-006) and the subject gave informed consent before the experimental sessions.

## CRediT authorship contribution statement

**Yota Kikuchi:** Conceptualization, Methodology, Visualization, Investigation, Writing – original draft. **Yasutomo Nomura:** Conceptualization, Writing – review & editing, Supervision, Resources, Funding acquisition, Project administration.

## Declaration of Competing Interest

The authors declare that they have no known competing financial interests or personal relationships that could have appeared to influence the work reported in this paper.

## Data Availability

No data was used for the research described in the article.

## Appendix Supplementary materials

Image, application 1

Image, application 2

Image, application 3

Image, application 4

Image, application 5
