# Supplementary material for: A working hypothesis visualization method for fNIRS measurements using Monte Carlo simulation
Source: MethodsX. 2023 Sep 9;11:102357. doi: 10.1016/j.mex.2023.102357 (PMC10506050; doi:10.1016/j.mex.2023.102357)
Supplement: Supplementary file 1 [file mmc1.docx]

**Supplementary Figure**

**
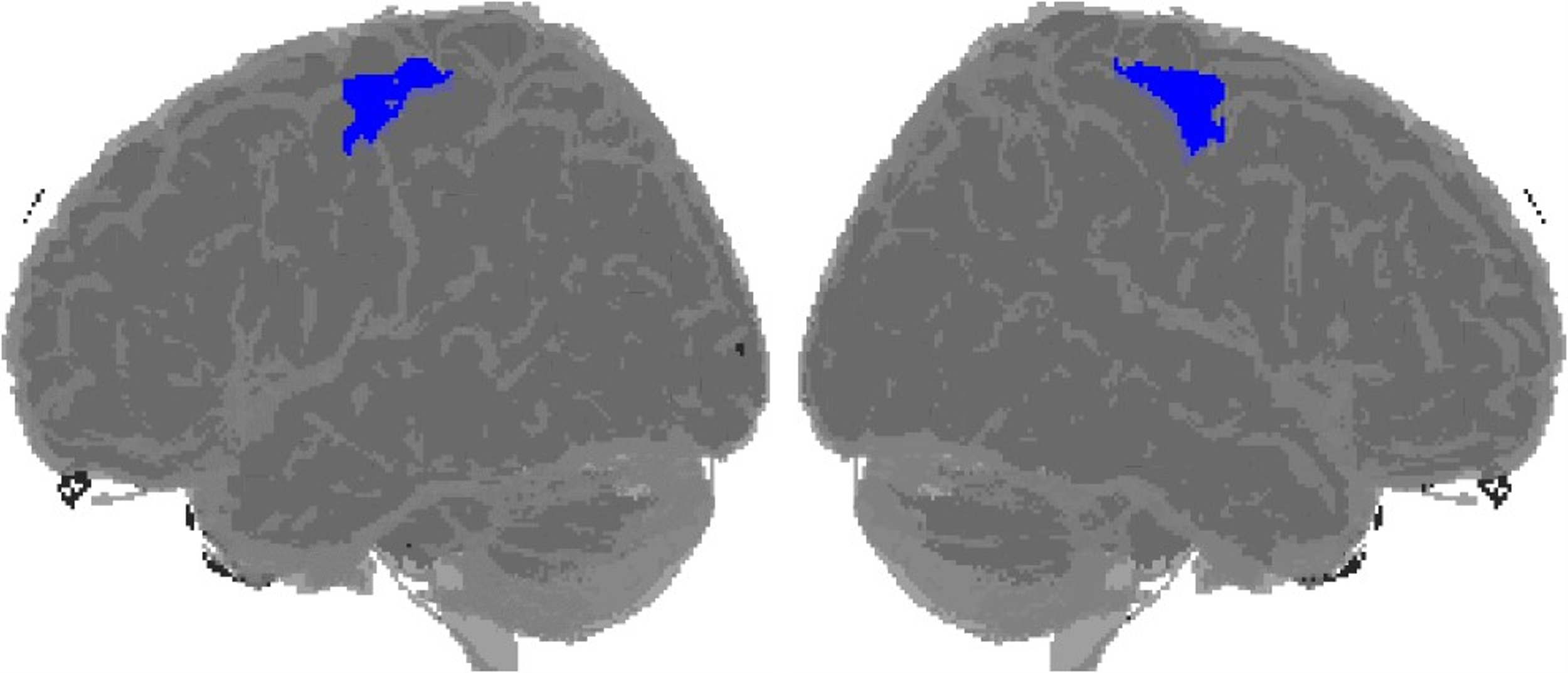
**

*Figure S1. Labeling results in the head voxel model. The left and right figures show the left and right cerebral hemispheres, respectively. The blue area was set as the ROI.*

**
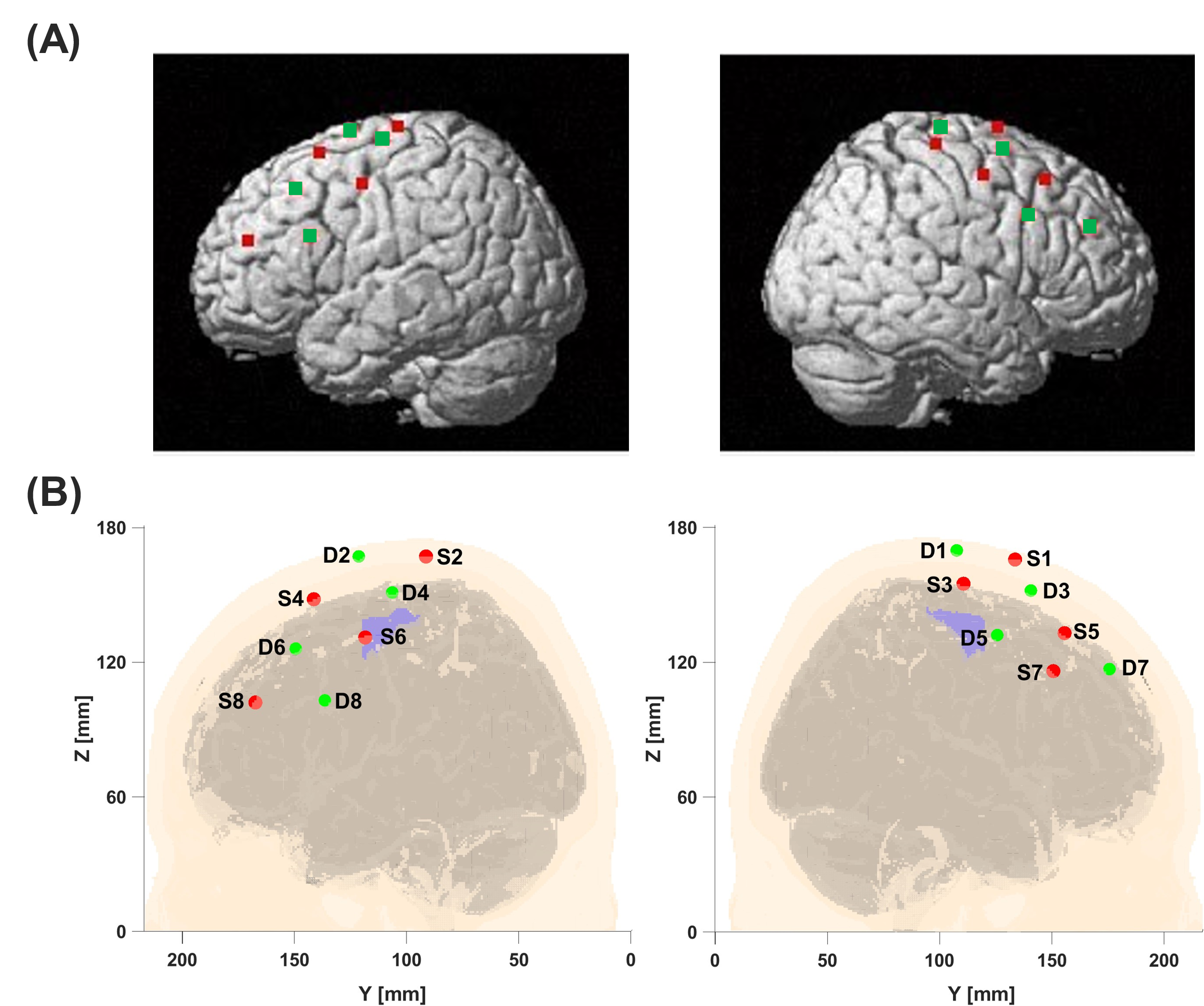
**

*Figure S2. Results of probe conversion. (A) Probes placed on the cerebral cortex of the MNI152 standard brain. (B) Probes converted on the scalp surface of the Colin27 head voxel model.*

**
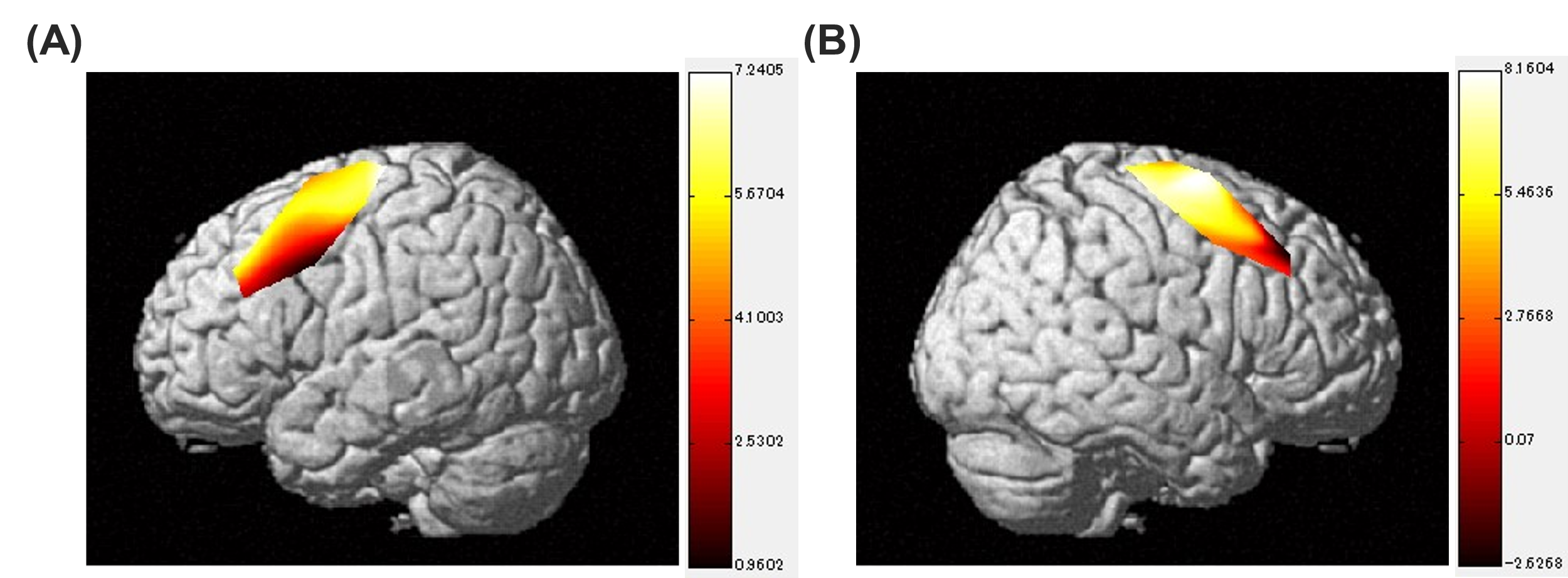
**

*Figure S3. T-statistical maps. (A) Result of the left cerebral hemisphere in right finger tapping. (B) Result of the right cerebral hemisphere in left finger tapping.*

**
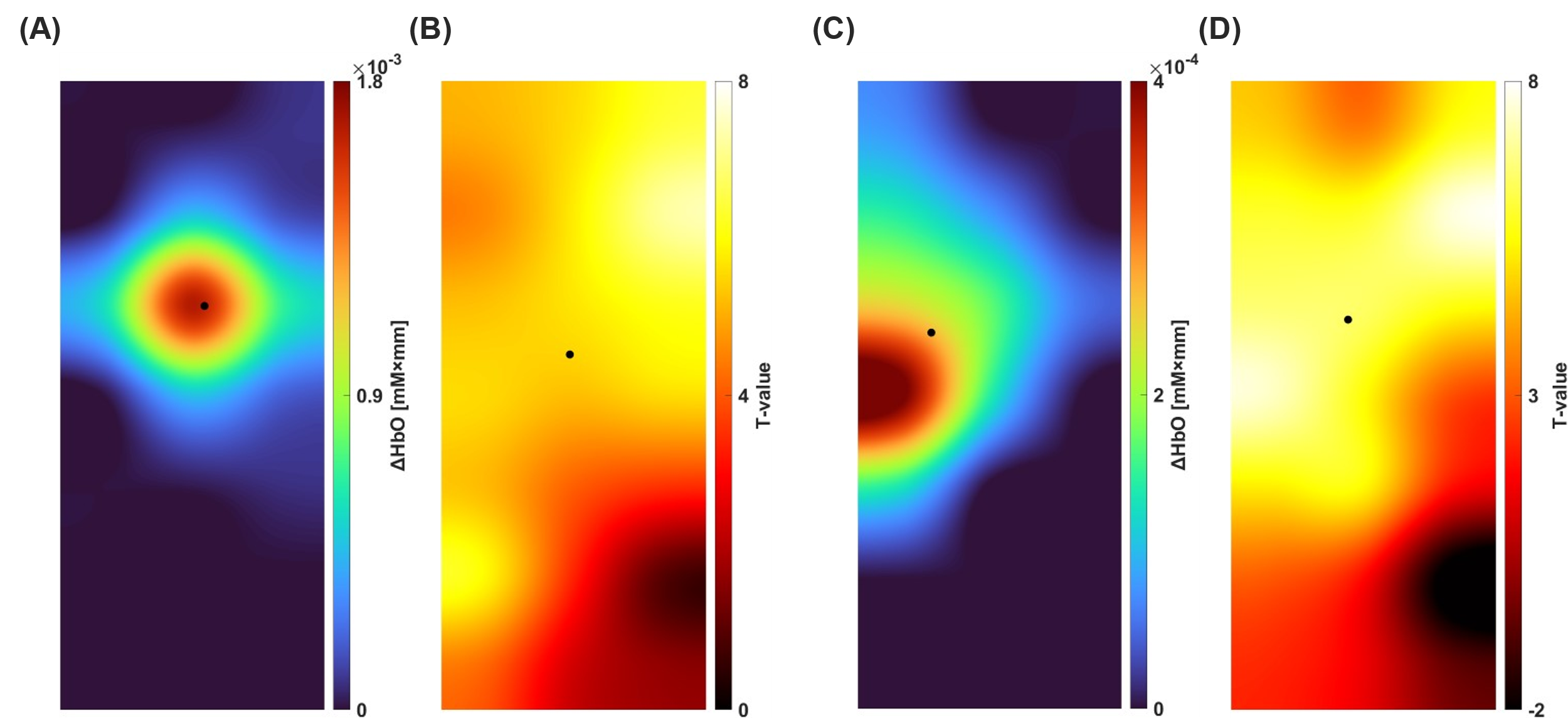
**

*Figure S4. The centroid of 2D activation maps. Left hemisphere activation map for right finger tapping in the simulation (A) and in the t-statistical analysis (B). Right hemisphere activation map for left finger tapping in the simulation (C) and in the t-statistical analysis (D). The black points indicate the centroid.*
